# Medical Device-Associated Biofilm Infections and Multidrug-Resistant Pathogens

**DOI:** 10.3390/pathogens13050393

**Published:** 2024-05-08

**Authors:** Nesrine Bouhrour, Peter H. Nibbering, Farida Bendali

**Affiliations:** 1 Laboratoire de Microbiologie Appliquée, Faculté des Sciences de la Nature et de la Vie, Université de Bejaia, Bejaia 06000, Algeria; nesrine.bouhrour@univ-bejaia.dz; 2 Department of Infectious Diseases, Leiden University Medical Center, 2300 RC Leiden, The Netherlands; nibbering@hhvbiotech.com

**Keywords:** nosocomial infections, biofilms, medical device, pathogens, bloodstream infections, urinary tract infections

## Abstract

Medical devices such as venous catheters (VCs) and urinary catheters (UCs) are widely used in the hospital setting. However, the implantation of these devices is often accompanied by complications. About 60 to 70% of nosocomial infections (NIs) are linked to biofilms. The main complication is the ability of microorganisms to adhere to surfaces and form biofilms which protect them and help them to persist in the host. Indeed, by crossing the skin barrier, the insertion of VC inevitably allows skin flora or accidental environmental contaminants to access the underlying tissues and cause fatal complications like bloodstream infections (BSIs). In fact, 80,000 central venous catheters—BSIs (CVC-BSIs)—mainly occur in intensive care units (ICUs) with a death rate of 12 to 25%. Similarly, catheter-associated urinary tract infections (CA-UTIs) are the most commonlyhospital-acquired infections (HAIs) worldwide.These infections represent up to 40% of NIs.In this review, we present a summary of biofilm formation steps. We provide an overview of two main and important infections in clinical settings linked to medical devices, namely the catheter-asociated bloodstream infections (CA-BSIs) and catheter-associated urinary tract infections (CA-UTIs), and highlight also the most multidrug resistant bacteria implicated in these infections. Furthermore, we draw attention toseveral useful prevention strategies, and advanced antimicrobial and antifouling approaches developed to reduce bacterial colonization on catheter surfaces and the incidence of the catheter-related infections.

## 1. Introduction

Every year, millions of catheters are implanted by health services to improve the management of acute and chronic diseases in adults and pediatric patients [1,2,3]. Unfortunately, their use inevitably allows patient’s own flora or accidental environmental contaminants to access the underlying tissues and cause fatal complications [4,5,6]. About 60 to 70% of nosocomial infections (NI) are linked to medical devices [7]. The main complication results from the ability of microorganisms to adhere to surfaces and form biofilms [8], which protects them and helps them to persist in the host [9].Indeed, biofilms act as a protection barrier against antimicrobial agents thereby leading to therapeutic failure and increased mortality and morbidity rates [3,10,11]. Moreover, in the biofilm, there is a small subpopulation called persister cells which are characterized by increased tolerance to antimicrobials.Once the antibiotic is removed, surviving persisters are able to re-grow causing infections [12]. Several studies demonstrated that persister cells are strongly involved in chronic infections and their recalcitrance in clinical, making the antibiotic treatment innefective and biofilm eradication impossible [13,14,15,16].

In fact, catheter associated bloodstream infections (CA-BSIs) are an important cause of hospital-acquired infections originating from an intravenous catheter and associated with morbidity, mortality, and hospital cost [17]. Central venous catheters (CVCs) are among the most widely used medical devices in critically ill patients. However, central line-associated bloodstream infections (CLA-BSIs) are the most common complications which are usually associated with the use of these CVCs [4], causing an increase in the rate of morbidity and mortality in health establishments as well as the length and costs of the stay [18]. In fact, 80,000 CVC-associated bloodstream infections (CVC-BSIs) mainly occur in intensive care units (ICUs) with a death rate of 12 to 25% [19]. Concerning peripheral venous catheters (PVCs), it has been estimated that 30 to 80% of hospitalized patients have a PVC in place during their hospitalization [20] and more than a billion PVCs are used each year around the world [21]. Among the side effects observed when a PVC is used are the following: phlebitis, partial dislodgement, accidental removal, occlusion, infiltration (fluid moving into surrounding tissue), and rarely, infections [22,23]. The incidence of bloodstream infections associated with peripheral venous catheters (PVC-BSIs) is generally low, with a rate of 0.1% of short catheters inserted (0.5 episodes per 1000 days of intravascular catheter) [24], unlike the incidence of CVC-BSIs which is 2.7 episodes per 1000 days of intravascular catheter [20]. Moreover, VC can be easily colonized by pathogenic microorganisms which lead to the formation of biofilms, other potential sources of BSIs [25]. Biofilms formed on CVCs were first described in 1982, during an epidemic of *Staphylococcus epidermidis* BSI [26]. Since that day, several studies have confirmed the involvement of biofilm in the pathogenesis of CVC-related infections and their importance [27]. Additionally, 81% of all vascular catheters that were placed in situ for 1–14 days were reported to be colonized by bacteria in the biofilm [28]. Biofilm colonization of intravenous catheters continues to affect healthcare settings [29]. Several factors increase the risk of catheter infections such as patient immunodeficiency, length of prolonged catheterization, catheter material, anatomical site of catheter insertion, poor hygiene, poor catheter insertion, and handling methods [30]. It has been reported that the incidence of bacteremia associated with PVCs is lower than that of bacteremia associated with CVCs. However, the duration of PVC insertion is 15 times longer than that of CVC insertion; for this, the number of PVC-BSIs is high due to the high number of patients who have a PVC [20,31].

Similarly, catheter-associated urinary tract infections (CA-UTIs) are the most commonly hospital-acquired infections worldwide [32]. These infections represent up to 40% of nosocomial infections. Also, 70% of UTIs are associated with urinary catheters (UC) and approximately 20% of hospitalized patients have a UC, especially those in ICUs [33]. Despite the high risk of acquiring infections with multidrug-resistant (MDR) opportunistic pathogens, most cases of catheter-associated bacteriuria are asymptomatic. However, when an episode of CA-UTI becomes symptomatic, the resulting sequelae can range from mild (fever, urethritis, and cystitis) to severe (catheter encrustation, bladder stones, pyelonephritis, endotoxic shock, and bacteremia). Left untreated, these infections can lead to urosepsis and death [34,35]. Indeed, for each day that a urinary catheter is in situ, there is a 3–8% incidence of bacteriuria, and in the majority of cases, long-term catheterization results in continued bacteriuria and symptomatic CA-UTI [36]. For an infection to be classified as a CA-UTI, a patient must have the following: (i) a urinary catheter implantated for more than 48 h; (ii) a symptom such as fever, pain, suprapubic tenderness, urinary frequency or urgency or dysuria; and (iii) urine culture with ≥10^5^ CFU/mL of a bacterial species [37]. However, there is much controversy over the CFU/mL cut-off in samples taken from a urinary catheter and several authorities consider that a number (greater than or equal to) ≥10^3^ CFU/mL is indicative of a true CA-UTI [38,39,40]. Moreover, other host factors such as female gender, older age (i.e., age > 50 years old), diabetes mellitus, faecal incontinence, immunocompromised status; healthcare factors such as lack of systemic antibiotics, catheter insertion outside the operating room, prolonging the duration of catheterization, and poor quality of catheter care [40,41,42] increase the risk of CA-UTIs. CA-UTI is linked with biofilm formation along the surface of the catheter [36]. Indeed, the presence of a UC facilitates bacterial colonization due to the development of a conditioning film of host proteins which provides bacteria with an ideal substrate for fixation [43].

In view of all this, there is an urgent need to develop novel strategies to fight medical device-associated biofilms. Despite several studies having been conducted in this field, many challenges still remain. This review provides an overview on the medical device-associated biofilm infections (mainly venous catheter-associated bloodstream infections and catheter associated-urinary tract infections), the biofilm development process on these devices, and the most MDR-bacteria implicated in these infections with their virulence factors. Furthermore, the current review highlights the different prevention strategies and the most effective approaches using antimicrobial coating and antifouling methods, to reduce medical device colonization and the incidence of their related infections.

## 2. Hospital-Acquired Infections

Hospital-acquired infections (HAIs) or nosocomial infections (NI) are defined as infections which were neither present nor incubating during the patient’s hospitalization and were acquired after 48 h of hospitalization. These infections increase patient morbidity and mortality, prolong their hospital stays, and represent a massive additional financial burden for health structures [44]. The severity of infection and its incidence is much higher in patients in burn units, intensive care units, organ transplant receivers, and with newborns due to their immunological status [45]. In addition to the problems associated with nosocomial infections, antibiotic resistance and the emergence of MDR-bacteria is a serious global problem, due to the uncontrolled administration of drugs [46]. These HAIs are often the result of the use of invasive procedures such as the location of temporary indwelling devices (VCs, UCs, endotracheal tubes, and wound drains) or are associated to the placement of cardiovascular or orthopedic implants during a surgical intervention [47]. They include a wide range of infections such as catheter related infections (CRIs), CA-UTIs, and ventilator associated pneumonia (VAP) [48]. These infections are generally designed as “Medical Device-Associated Biofilm Infections” [3]. Several Gram-negative (*Escherichia coli*, *Klebsiella pneumoniae*, *Proteus mirabilis*, *Pseudomonas aeruginosa*, and *Acinetobacter baumannii*) and Gram-positive (*Staphylococcus aureus*, *Staphylococcus epidermidis*, and *Enterococcus faecalis*) bacteria are involved in the onset of NIs [49,50]. Their ability to form a biofilm makes the treatment of these infections more complicated [51].

In fact, it is well known that biofilms have a significant impact in medicine through the development of HAIs [52] and it is estimated that bacterial biofilms are involved in 65% of NIs and in more than 80% of chronic infections [53]. Treatment of these infections requires administration of high dose antibiotics and/or replacement of the device, which are both ineffective due to the antibiotic resistant strains and the high risk of re-infection on the new device [51].

## 3. Biofilm Formation on Medical Devices

**Figure 1:**
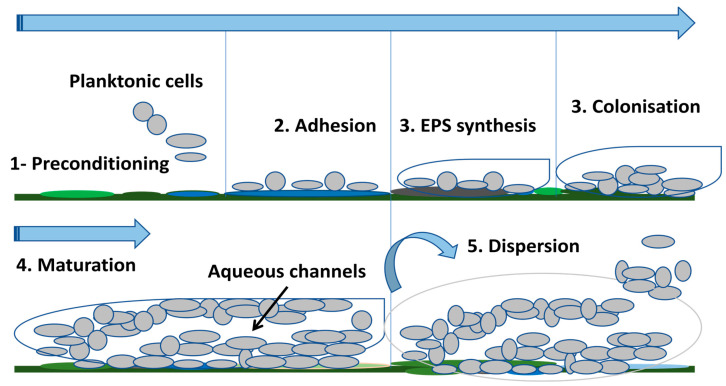
Biofilm formation steps on medical devices.

Bacteria have always been studied in the laboratory as planktonic microorganisms. However, most bacteria live in multicellular communities called biofilms [54]. The biofilm was observed for the first time in the 17th century by Anthony van Leeuwenhoek through his microscope in his own mouth where he observed aggregated microorganisms on his teeth and tongue [55]. A biofilm is a highly structured bacterial community attached to a surface and protected by a self-produced extracellular polymeric matrix [56,57,58]. This matrix is mainly composed of proteins, polysaccharides, and extracellular DNA (eDNA). Furthermore, the biofilm matrix is highly hydrated and contains up to 97% water, mainly responsible for nutrient transport within the biofilm [53,59]. Bacterial adhesion occurs on a pre-conditioning film formed on the surface after deposition of organic, inorganic, and cellular components (e.g., fibronectin, fibrinogen, laminin, collagen, polysaccharides) found in the environment surrounding the medical device, constituting a base on which the biofilm will develop [51,57]. The bacteria interact with the components of this surface through appendages, attractive forces, or adhesins [57]. The biofilm formation on a medical device proceeds as follows: (i)Transport of bacterial cells to the surface—the bacteria can be transported to the medical device either by diffusion (Brownian motion), convective flow or active movement (motile bacteria) [60]. Bacterial transport can also be induced by chemotaxis due to the presence of diffusible chemical gradients which form from various chemical stimuli or the degradation of components (eg. aspartate, glucose, galactose) [60,61]. However, if the surface is unsuitable for bacterial adhesion, the bacteria return to the planktonic state [11]. (ii) Reversible attachment—this first step of the bacterial adhesion process to the medical device is the transport of cells to the device [61]. The initial attraction mainly involves non-specific physical interactions such as Van der Waals attractive forces, electrostatic forces (attractive or repulsive), hydrophobic interactions, Brownian motion, and gravitational forces [62]. Furthermore, due to the negative charge of their cell membrane, bacteria are subjected to repulsive electrostatic and repulsive hydrodynamic forces when they are near the medical device. In order to overcome these two repulsive barriers, bacteria typically use cellular appendages, such as flagella or pili [63,64]. This initial binding of bacteria to the medical device is important to make irreversible adhesion possible [60]. (iii) Irreversible attachment—this step is characterized by stronger induced cell–surface interactions and shorter distances which allow adhesins exposed to the cell surface to form a bond with the biomaterial [65]. Gene expression which encodes for bacterial surface structures including fimbriae, pili, lipopolysaccharides, and slime will also begin to strengthen adhesion promoting biofilm formation [65,66]. (iv) Cell proliferation and formation of microcolonies:—once the bacteria have become attached to the surface of the medical device and stabilized, the cells will proliferate rapidly and produce intercellular adhesins to form microcolonies. In this step, the QS “quorum sensing” communication system is activated when the bacterial density reaches a threshold [11,57,67]. The gene expression of components required for a biofilm matrix such as polysaccharides, proteins, eDNA, and lipids is also activated [68]. (v) Device surface colonization—the cells continue to proliferate and additional surrounding planktonic cells are also incorporated into the biofilm [69]. (vi) Biofilm maturation—the multilayers continue to form, inducing an increase in thickness, thereby allowing the transition from a two-dimensional arrangement to a three-dimensional arrangement (mature biofilm) [67] also called “mushroom” structure [70]. Channels filled with water are formed in the biofilm allowing the transport of nutrients, signaling molecules, and elimination of waste [53,71]. (viii) Biofilm dispersion—once the biofilm is mature, the planktonic bacteria detach from the biomaterial due to hydrolase enzymes then migrate and colonize new surfaces, spreading the infection [11,12]. The main steps are illustrated in Figure 1 below.

## 4. Most Common Pathogenic Bacteria Involved in Medical Device-Associated Biofilm Infections

### 4.1. Escherichia coli

*Escherichia coli* is responsible for a wide variety of community and HAIs such as UTIs and BSIs with increasing antimicrobial resistance rates [72,73]. Indeed, in addition to the global emergence of resistance to carbapenems, the significant increase in the prevalence of quinolones-resistant *E. coli* strains has also been reported in several countries limiting the treatment choice for these bacterial infections, thereby constituting a real problem for public health [74,75]. Uropathogenic *E. coli* (UPEC) is the bacterium mostly involved in 80 to 90% of UTI cases [76,77] and in 40% of hospital acquired UTIs [78]. These strains have become more resistant to antibiotics with the increasing prevalence of extended-spectrum β-lactamases (ESBLs) [74,79]. *E. coli* is one of the bacteria most implicated in biofilm-related infections, particularly in CA-UTIs [80,81,82,83,84] and CVC-BSIs [85,86,87,88]. In fact, biofilm formation is the major cause of these infections in catheterized patients, making them hard to eradicate [89,90]. Indeed, their ability to form a biofilm is associated with the persistence and chronicity of inflammations leading to complicated and/or recurrent infections [91,92]. *E. coli* strains have an arsenal of virulence factors which contribute to adhesion, colonization, and persistence allowing the different defense mechanisms of the host to be overcome [93]. Among them, we distinguish adhesins, *fimbriae*, toxins, siderophores, etc. Table 1 summarizes the virulence factors implicated in *E. coli* pathogenesis and especially adhesion and biofilm formation. The role of virulence genes of *E. coli* in its adhesion to catheter surfaces has been reported in several studies [82,83,89,90,92,94,95,96,97,98,99,100,101,102]. For example, Reisner et al. [94] reported that 73% of *E. coli* strains isolated from catheterized patients expressed type 1 *fimbriae*. In a recent study of Zou et al. [90], they showed that biofilm associated genes such as iron transport systems (ferric citrate) and antigen 43 may be involved in the pathogenic CA-UTI strains. Another recent study demonstrated that the knockout of *luxS*, *fimH*, and *bolA* genes decreased EPS matrix production, which is very important in *E. coli* biofilm-associated UTIs [83].

### 4.2. Klebsiella pneumoniae

*Klebsiella pneumoniae* is considered to be one of the most important opportunistic pathogens responsible for NIs including sepsis, soft tissue infections, pneumonia [168], and UTIs, which are the most common worldwide [169]. *K. pneumoniae* is implicated in 6–17% of opportunistic UTI cases mainly linked to bacterial adhesion in the inner and outer surfaces of the urinary catheter [170,171]. Moreover, it is the second pathogen involved in BSIs, after *E. coli* [172]. The prevalence of NIs due to *K. pneumoniae* has been reported to be approximately 10% worldwide [173]. Several studies revealed the implication of *K. pneumoniae* in HAIs, especially in catheter-related infections (CRIs) [18,85,172,174,175,176,177,178,179]. Since this bacterium is commensal in humans, gastrointestinal colonization represents the major source of transmission and the development of infections towards other sites [180]. The global antibiotic resistance rate of *K. pneumoniae* is approximately 70% with mortality rates ranging from 40% to 70% [181] and the emergence and spread of MDR-strains of *K. pneumoniae* have becomea real global problem [182]. In fact, this bacterium is able to acquire resistance genes such as ESBLs or carbapenemases (resistance to cephalosporins or third-generation carbapenems), limiting treatment options for infections [168], causing serious or even fatal infections, while also increasing the length of hospitalization and the costs of processing [183]. Moreover, carbapenem-resistant *K. pneumoniae* are the major cause of BSIs with high rates of mortality and morbidity in the world [179]. Another group of *K. pneumoniae* strains called “hypervirulent”, able to express acquired virulence factors, has also emerged causing serious community-acquired infections [184]. Many *K. pneumoniae* isolates are able to form biofilms, resulting in increased impermeability to antibiotics causing treatment failure [185]. For this, the World Health Organization (WHO) classifies this bacterium among the high priority species and encourages the development of new antimicrobial molecules in order to counter its antibiotic resistance [169]. *K. pneumoniae* has several virulence factors, including types 1 and 3 *fimbriae*, capsule polysaccharides, LPS, *quorum-sensing*, and PGA [118] used to escape host immune defenses, biofilm formation, and to persist during infection [186].The adhesins of *K. pneumoniae* allow the establishment of strong biofilms [116,187,188,189,190,191,192,193,194].An in vitro study reported the improvement of biofilm formation on UCs by the presence of types 1 and 3 *fimbriae* in a bladder model [116]. In a recent study, the results showed that 87.5%, 46.4%, and 53.6% of strains harbored *fimH*, *mrkA*, and *mrkD* fimbrial genes, respectively [193]. Other genes have been reported to be involved in biofilm formation [119,187,191,194,195,196,197]. The main virulence factors of *K. pneumoniae* are summarized in Table 1.

### 4.3. Proteus mirabilis

*Proteus mirabilis* is an opportunistic pathogen that causes infections in immunocompromised individuals but also NIs, including wound infections, blood infections, and mainly UTIs [50]. It is recognized as the main cause of CA-UTIs, and in the USA, 3% of all HAIs and 44% of CA-UTIs are linked to this bacterium [126]. In fact, *P. mirabilis* is considered the third most common cause of UTIs and the second most common cause of CA-UTIs in long-term catheterized patients [198]. Furthermore, UTIs, particularly CA-UTIs, caused by *P. mirabilis* generate serious complications such as the formation of bladder and kidney stones, permanent kidney damage, or even bacteremia/sepsis which can be fatal for patients [199]. In addition, trauma to the urethra and bladder mucosa may also occur during catheter removal [124]. All these complications are due to the capacity of *P. mirabilis* to form crystalline biofilms, leading to an obstruction [126]. *P. mirabilis* was reported as a common agent causing UTIs and CA-UTIs in several studies [122,126,198,200,201,202,203]. This commensally bacterium colonizing the perianal area of patients, easily penetrates into the bladder after implantation of the catheter, and thus adheres to it [204]. In addition, in recent years, *P. mirabilis* strains have become increasingly resistant to drugs, especially, the isolates producing ESBLs, leading to therapeutic failure, thereby constituting a worldwide problem for public health [123,205,206,207].

*Proteus mirabilis* has a panel of virulence factors (Table 1); however, its pathogenicity is exacerbated by biofilm formation, making the infection worse [208]. In fact, this uropathogen has been shown to have a great ability to form biofilm and is involved in the encrustation and blockage of UCs—a common complication in patients with long-term indwelling urinary catheterization—and is a major cause of morbidity and mortality in CA-UTIs [124,129]. Numerous adhesins, including MR/PM *fimbriae*, MR/KH, PM *fimbriae*, urethroepithelial adhesin (UCA), and ambient-temperature *fimbriae* (ATF) have been associated to *P. mirabilis* adhesion onto UCs and biofilm formation during CA-UTIs [126,203,209,210,211].

### 4.4. Pseudomonas aeruginosa

*Pseudomonas aeruginosa* is one of the six bacterial pathogens of the ESKAPE group (*Enterococcus faecium*, *Staphylococcus aureus*, *K. pneumoniae*, *Acinetobacter baumannii*, *P. aeruginosa*, and *Enterobacter* species) known for their high antibiotic resistance and increased virulence, which pose big challenges to treatment worldwide [136,212,213]. In fact, the resistance of this pathogen to many antibiotics, particularly resistance to carbapenems, constitutes a serious threat to global public health and increases morbidity and mortality rates, particularly in ICUs [137,214]. This is why the WHO designated this MDR-bacterium as one of the priority antibiotic-resistant pathogens for which new antibiotics are urgently needed [136]. *P. aeruginosa* is one of the most opportunistic pathogens causing fatal acute or chronic infections in immunocompromised hosts. Indeed, it is one of the most common pathogens found in hospitals and is responsible for more than 50% of NIs [213]. The most HAIs caused by *P. aeruginosa* are VAP, UTIs, CA-BSIs, burn wound infections, skin and soft tissue infections, surgical site infections, and ocular infections [131,215]. Moreover, *P. aeruginosa* has the ability to form biofilms during infections causing increased resistance to antibiotics and the persistence of NIs, which are considered fatal for patients [216]. It is estimated that *P. aeruginosa* is responsible for 28% of device-related infections [217]. A rate of 11.5% of *P. aeruginosa* strains was recovered from UCs and all of them were MDR [217]. The implication of *P. aeruginosa* in CA-UTIs [218,219,220,221] and in CL-BSIs [85,87,88,175,222] has been widely reported. Importantly, a recent international study on infections in ICU-patients demonstrated that *P. aeruginosa* was responsible for 23% of all infections acquired in ICUs, with the respiratory source being its main site [223]. These bacteria have an impressive arsenal of virulence factors which contributes to their pathogenesis (Table 1). Flagella and pili are virulence factors involved in motility as well as bacterial adhesion, which is the starting point of infections [214,224,225,226,227]. Also, the EPS Psl and Pel, major components of the biofilm matrix of *P. aeruginosa*, significantly contribute in bacterial adhesion to the catheter surface, cell–cell aggregation, and stability of the biofilm structure [217]. Several reports showed the importance of these EPS [219,228,229].

### 4.5. Acinetobacter baumannii

*Acinetobacter baumannii* is a nosocomial pathogen that is responsible for a large number of infections in humans, including endocarditis, UTIs, meningitis, pneumonia (in mechanically ventilated patients), and sepsis [141]. The incidence rate of *A. baumannii* infections is estimated to be approximately one million cases per year in the world, with high mortality rates, especially in critically ill patients [230]. The high prevalence of MDR-*A. baumannii* has become a serious situation in a hospital setting. One of the major factors responsible for the chronicity and persistence of infections and resistance to antibiotics of *A. baumannii* is its ability to colonize and form a biofilm on biotic and abiotic surfaces (e.g., vascular catheters, cerebrospinal fluid shunts, or Foleys catheter) [231,232]. In addition to MDR-*A. baumannii*, extremely-drug resistant (XDR) and pan-drug resistant (PDR) isolates have also been reported worldwide [230]. Most *A. baumannii* infections occur in ICU-patients and account for up to 20% of ICU-infections worldwide [141,233], with an increase in mortality rates (30% to 75%) [234]. Moreover, their ability to acquire resistance to antibiotics and persistence in the environment are the main factors contributing to their survival in the hospital environment [235]. CRIs associated with MDR-*A. baumannii* biofilms have been widely reported [236,237,238,239,240,241,242,243]. *A. baumannii* possess several adhesins (Table 1), which contribute to biofilm formation and bacterial colonization on medical devices [230]. Several reports have shown the presence of *omp*A (detection from 81 to 100%), *csuE* (detection from 80 to 100%), and *bap* (detection from 43 to 91.4%) in clinical *A. baumannii* strains producing strong biofilms [232,244,245,246,247,248]. In a recent study, Kasperski et al. [249] reported that 72% of strong biofilm producer *A. baumannii* strains harbored four genes associated with biofilm formation (*bap*, *bfmS*, *csuE*, and *ompA*), showing their implication in bacterial adhesion on surfaces.

### 4.6. Staphylococcus aureus

*Staphylococcus aureus* is a clinical pathogen that causes infections in both humans and animals, ranging from mild infections to severe invasive life-threatening infections [250]. This organism is also the main agent involved in NIs due to its incredible ability to adhere to the surface of medical devices and form a biofilm, which often leads to chronic infections such as osteomyelitis, endocarditis, CF, catheters infections, prostheses infections, and other medical device-associated infections [251,252,253]. *S. aureus* is one of the major pathogens responsible for nosocomial blood infections with an incidence estimated between 10–30 cases/100,000 persons/year and associated with mortality rates ranging between 15 and 40% [254]. In addition to this, the global emergence of methicillin-resistant *S. aureus* (MRSA) is a major public health concern given its high virulence and therefore, treatment failure is unavoidable [155], increasing mortality and morbidity in patients [251]. It is important to note that the *mecA* gene is the genetic determinant of methicillin resistance in MRSA, which is located on the mobile genetic element called staphylococcal cassette chromosome mec (*SCCmec*) [255]. It has a remarkable arsenal of virulence factors (Table 1) including toxins, proteases, nucleases, but also many proteins anchored in the cell wall which important factors are allowing it to adhere to tissues, to surfaces, to form a biofilm, and to escape the host’s immune defense [256,257]. It has been reported that the mortality rates associated with MRSA bacteremia were higher than those associated with methicillin-sensitive *S. aureus* (MSSA) bacteremia [258]. CR-BSIs due to *S. aureus* are considered to be the most fearful of infections. Indeed, Mandolfo et al. [259] identified 113 CR-BSIs caused by MRSA (47.5%) and MSSA (52.5%) in hemodialysis patients. In the study of Bonnal et al. [260], 56% of *S. aureus* in PVC-BSIs and 34% of *S. aureus* in CVC-BSIs were identified. Recently, Pinto et al. [261] identified *S. aureus* as the main causative agent of CR-BSIs with a rate of 24.1%. Other studies [85,175,222,258,262,263,264,265,266] also reported implication of *S. aureus* in CR-BSIs. *S. aureus*, especially MRSA, also constitutes a serious problematic pathogen in CA-UTIs [267]. In fact, *S. aureus* can colonize the urinary tract via urinary catheterization causing ascending UTIs. The presence of MRSA complicates the situation, extending the length of hospital stay [268]. This pathogen causes approximately 0.2–4% of UTIs and is more often found in patients in long-term care and with long-term catheters [269]. Several studies have shown the implication of *S. aureus* in CA-UTIs [267,270,271].

Various reports showed the implication of virulence genes in *S. aureus* healthcare infections including CRIs. The major factor in staphylococcal biofilm formation is the polysaccharide intercellular adhesin (PIA) or poly-N-acetyl β-1-6 glucosamine (PNAG), encoded by the *icaADBC* operon [272,273,274]. This polysaccharide plays an important role in colonization, biofilm formation and biofilm-related infections, antimicrobial resistance, immune evasion, and phagocytosis [146]. Several studies reported a positive correlation between the presence of *icaAD* genes and the ability of *Staphylococcus* strains to produce a biofilm [275,276,277]. However, Pinto et al. [261] showed no relationship between the presence/absence of the *ica* operon and biofilm formation on CVCs. Another recent study showed that 4/6 *S. aureus* strains, that did not carry *ica* genes, were strong biofilm producers [278]. This indicates that other genes may be involved in biofilm formation. Indeed, *S. aureus* contains also a range of proteins categorised as “microbial surface component recognising adhesive matrix molecules (MSCRAMM)” [146,149] such as fibronectin-binding proteins (FnBPA and FnBPB), clumping factors (ClfA and ClfB), collagen adhesin (Can), elastin binding protein (EbpS), fibrinogen binding protein (Fib), laminin-binding protein (Eno), and serine aspartate repeat proteins C, D, and E (SdrC, SdrD, SdrE) [279,280], which have been implicated in binding host matrix components (fibronectin, fibrinogen, collagen) to initiate cell attachment and/or biofilm formation [150]. The association between the MSCRAMMs adhesins ClfA/B, FnbA/B, and Cna with bacteremia and catheter-related bacteremia has also been reported [281,282]. Similarly, Walker et al. [267] reported that the clumping factor ClfB, interacting with fibrinogen, facilitates colonization of the UC and bladder leading to infection in mice and humans in a CA-UTI model.The expression of these virulence factors and biofilm formation are regulated by global regulatory systems such as accessory gene regulator (*agr*), staphylococcal accessory element (*sae*), and also by staphylococcal accessory regulator A (*sarA*) [155]. Pérez-Montarelo et al. [283] reported that more than half of MRSA-related bloodstream isolates belong to the accessory gene regulator (*agr*) group II.

### 4.7. Staphylococcus epidermidis

*Staphylococcus epidermidis* is one of the most ubiquitous opportunistic pathogens [284] that causes serious infections in immunocompromised patients, especially those associated with the presence of invasive medical devices (e.g., VC and artificial heart valves) [156]. However, this bacterium rarely causes CA-UTIs and rarely pyelonephritis (without an indwelling urinary device) [285]. It is estimated that 30% of CA-BSIs are due to *S. epidermidis* strains [286]. *S. epidermidis* isolates were implicated in several CA-BSIs. A recent report revealed that this species was the most causative agent in CA-BSIs (13.3%) in the emergency department [287]. A prospective observational study conducted by Pinto et al. [261] showed that *S. epidermidis* was the most etiological agent of CR-BSI. A similar retrospective study on CA-BSI in coronavirus disease 2019 ICU showed also the prevalence of *S. epidermidis* [288]. Other prevalence rates have been also reported: 31.37% [289], 31% [290], 28% [291], 18.1% [292], 12.33% [286], 8.3% [293], and 7.7% [261]. The European Center for Disease Prevention and Control reported that *S. epidermidis* caused 23.6% of CA-BSI cases in ICUs [261]. It has also been reported that children are very susceptible to infection associated-methicillin-resistant *S. epidermidis* strains in perinatal units [294]. In addition, in recent years, the situation has become complicated with the capacity of *S. epidermidis* strains to form biofilms on medical devices and their increased resistance to antibiotics, mainly methillin resistance, leading to significantly high mortality and morbidity rates and medical costs [295,296]. It has been reported that more than 70% of *S. epidermidis* strains are resistant to methicillin, which is encoded by *mecA* gene, making the treatment of infections ineffective but above all that this resistance to methicillin can be quickly disseminated to other Gram-positive strains via horizontal gene transfer [297]. *S. epidermidis* strains produce a variety of virulence factors contributing to their pathogenicity (Table 1). However, unlike *S. aureus*, *S. epidermidis* isolates are weakly virulent, do not produce aggressive toxins, and are commonly non-hemolytic [159]. The main virulence factor is its ability to adhere to the surface of medical devices and form a biofilm [298,299]. During accumulation, *S. epidermidis* produces a major component of the biofilm matrix which is PIA [156]. As for *S. aureus*, PIA, encoded by *ica* opeon, is an essential factor in *S. epidermidis* biofilms allowing their adhesion to surfaces [300]. Cherifi et al. [301] found that the *ica* operon was significantly more present in CR-BSI isolates than in commensal isolates. However, François et al. [274] reviewed that the expression of *ica* operon is not essential in the colonization of a surface, and the presence of other virulence genes such as *atlE*, *fbe*, and *embp* are involved in catheter-related infections linked to *S. epidermidis* biofilms [286] The pathogenesis of *S. epidermidis* is regulated by two key systems, the *agr* and the *sar* regulators, which allow the expression or repression of the virulence genes in a coordinated manner during infection [160]. An early study showed that the *agr QS* system played an important role in the long-term development of *S. epidermidis* biofilm during medical device-associated infections [302].

### 4.8. Enterococcus *spp.*

Enterococci are Gram-positive, non-motile, lactic acid-producing bacteria widely found in the gut microbiota of humans and animals [303]. Enterococci are facultative anaerobes that tolerate a variety of environmental conditions such as extreme pH, salinity, and a wide temperature range (10 to >45 °C) [163]. Enterococci are tenacious microorganisms characterized by increased tolerance to desiccation and starvation, making them resistant to environmental stresses [304]. The emergence of *Enterococcus* strains as HAIs agents [305] can be explained by the following: (i) these bacteria are intrinsically resistant to several classes of antibiotics (cephalosporins, macrolides, clindamycin, and trimethoprim–sulfamethoxazole) and they acquired resistance to ampicillin, ciprofloxacin, high-level aminoglycosides, and vancomycin [306], causing a serious problem for the treatment of these infections given the limited choice of available antibiotics (linezolid, daptomycin, quinupristin/dalfopristin, and vancomycin) [307]; (ii) they have an impressive capacity to acquire new resistance genes due to the plasticity of their genome [308].

*Enterococcus* spp., particulary *En. faecalis* and *En. faecium*, are the most opportunistic pathogens causing several infections, including medical device-associated infections, UTIs, wound infections, and BSIs [308]. *En. faecalis* is responsible for 80 to 90% of cases of Enterococci-associated NIs, followed by *En. faecium* (5 to 10% of infections) [309,310]. Putta et al. [311] revealed that *En. faecalis* was the most causative agent of CA-UTIs in ICU-patients. In another study, 13.11% of *En. faecium* was isolated from CA-UTIs [312]. Different rates were obtained in other studies: 91% [313], 29% [84], 22.9% [314], 19% [315], and 7.1% [316]. The presence of these pathogens in CL-BSIs was also reported [317,318]. *En. faecalis* was the 5th most frequently isolated bacteria from CA-UTIs and the 3rd from CLA-BSIs, unlike *En. faecium*, which was the 11th and 5th in CA-UTIs and CLA-BSIs, respectively [303]. However, in recent years, the prevalence of infections due to *En. faecium* has increased, overtaking the prevalence of *En. Faecalis*, and this is due to the emergence of antibiotic resistance, especially vancomycin-resistant *En. faecium* (VREfm), which is responsible for the most vancomycin-resistant *Enterococcus* (VRE) infections in the world [319,320,321], thus leading the WHO to include VREfm in the list of high priority pathogens [321].Of note, glycopeptides resistance in Enterococci is attributed to the acquisition of different clusters of genes (e.g., *vanA*, *vanB*, *vanD*, *vanE*, *vanG*, and *vanL*) which confer resistance [322,323]. In addition to antibiotic resistance, the ability of *Enterococcus* strains to form biofilms is one of the primary factors involved in their virulence and pathogenicity notably on medical devices [324,325]. Several investigations have been conducted on Enterococci virulence factors involved in biofilm formation. Soares et al. [326] found that *En. faecalis* possessed *esp*, *gelE*, and *asa1* genes. Kafil and Mobarez [327] reported the presence of *esp*, *ebpA*, and *ebpB* genes found in high biofilm producers. Similar results were found in the study of Khalil et al. [325]. It has been reported also that the endocarditis and biofilm-associated (Ebp) pilus is involved in biofilm formation on UC leading to CA-UTI [327,328]. However, in presence of urine, Ebp is not capable to initiate *En. faecalis* adhesion on UC. This fact was explained by the release of fibrinogen covering the catheter following an inflammatory response caused by the catheter itself [327]. In another study, the analysis of mutations affecting two proteases, secreted by *En. faecalis* (GelE, SprE), revealed that loss of both factors resulted in decreased CA-UTI and defective biofilm establishment in a murine CA-UTI model, whereas the loss of either had no effect. They revealed also that the high expression of these proteases depends on the *fsr QS* system [329]. Another study reported the importance of Ace and Esp adhesins in the bacterial attachment on the catheter surface, and biofilm accumulation [330].Given that *En. faecalis* is the most identified species in biofilm-associated infections [331,332], the virulence factors cited in Table 1 only concern this bacterial species.

## 5. Pathogenesis of Venous Catheter Contamination and Catheter-Associated Bloodstream Infections (CA-BSIs)

After insertion of a VC, the surface of the device is immediately covered by a conditioning film composed of organic molecules such as fibronectin and fibrinogen, collagen, elastin, and laminin [27,333]. There are several pathways involved in catheter contamination: colonization of the surface of the catheter tip by skin microorganisms originating from health care worker’s hands, contaminated disinfectant, which migrates to the insertion site in the skin pathway of the catheter and along the catheter surface (extraluminal) which is the most common route of infection for short-term catheters (inserted for ≤14 days), colonization of the inner surface of the catheter by contaminated infusion product and catheter hub (intraluminal), and finally, by hematogenous seeding (rare route) [17,334]. In contact with blood, microorganisms interact with fibrin to produce an adherent biofilm which will promote bacterial colonization and the spread of these microorganisms [334].

Colonization and biofilm formation on the catheter surface occurs 24 h after device insertion [335]. These pioneer bacteria allow the attachment of other pathogens by providing more diverse adhesion sites. After multiplication, the bacteria produce an extracellular matrix which maintains the biofilm, leading to irreversible adhesion to the surface of the catheter [27]. Once the biofilm is mature, bacteria can disperse, cause catheter associated bloodstream infections (CA-BSIs), and colonize other sites in the body [336].The formation of fibrin sheaths is also observed. Once the catheter is implanted, the fibrinogen, albumin, lipoproteins and coagulation factors, released due to the lesion of the blood vessels, begin to deposit on the surface of the catheter within 24 h forming a fibrin sheath which covers the surface of the catheter within days or even weeks. This fibrin sheath is responsible for the late stage catheter dysfunction, which usually occurs about three months after the catheter placement [333,337].

## 6. Pathogenesis of Urinary Catheter Colonization and Catheter-Associated Urinary Tract Infections (CA-UTIs)

Transurethral ascension of microorganisms is the most common mechanism for the development of UTIs, which explains the increased risk of infection after catheterization [338]. Bacteria can colonize UCs either by the endoluminal route which involves exogenous flora originating from colonization of the collecting sac or from a breach of the closed system during manipulations of the urinary catheter, or by the exoluminal route which involves the endogenous flora of the urinary meatus and occurs early during catheter placement or later following colonization of the urinary meatus by the digestive flora [6]. After insertion of the catheter, the bacteria overcome first the electrostatic repulsion observed between bacterial cell and catheter surface to allow intimate interactions to occur, then adhere to a conditioning film of urine components and host proteins, such as Tamm–Horsfall protein, magnesium and calcium ions, which form along the catheter surface. It is also reported that the urinary catheter elicits an inflammatory response resulting in the release of the host protein fibrinogen into the lumen of the bladder which will cover the surface of the catheter [330].Pathogens such as *S. aureus* and *Enterococcus faecalis* possess adhesins such as the fibronectin-binding protein A (FnBPA) and the endocarditis and biofilm-associated pilus (EbpA), respectively, which bind to the host fibrinogen in order to disrupt blood clotting, initiate biofilm formation, as well as immune evasion [339,340]. Once irreversibly fixed to the surface of the catheter via adhesins and pili, bacteria begin to change their phenotype, producing exopolysaccharides which protect them and form a biofilm [106,341]. The presence of biofilms promotes the appearance of epithelial lesions due to the proteases and bacterial toxins produced. The uropathogenic bacteria can then ascend to the kidneys, attaching again to the renal epithelium, causing kidney infections. Left untreated, these infections can progress to bacteremia by crossing the tubular epithelial cell barrier into the bloodstream [341].

## 7. Prevention of CA-BSIs

### 7.1. Education, Training and Surveillance

The lack of knowledge and skills is one of the main obstacles to medical practice. Indeed, compliance with guidelines for the use of intravascular catheters is very important in order to decrease the incidence of CA-BSIs and their associated health costs. Educating healthcare personnel regarding techniques for using intravascular catheters, the procedures for inserting and maintaining intravascular catheters, periodically assessing their knowledge and ensuring appropriate levels of nursing staff in intensive care units are a first line of prevention [342,343]. In addition, another effective measure to reduce CA-BSIs is to avoid unnecessary catheterization of patients as well as the rapid removal of venous catheters which are no longer necessary, particularly long-term catheters [344].

### 7.2. Aseptic Techniques

Hand hygiene before handling catheters, disinfecting catheter sites, catheter hubs or injection ports with an appropriate agent before accessing the catheter are essential for the prevention of CA-BSIs [345]. The use of 2% chlorhexidine–alcohol as an antiseptic agent before insertion of a VC and during dressing changes is recommended to prevent the development of CA-BSIs. The incidence of CA-BSIs was shown to be five times lower using 2% chlorhexidine–alcohol solution, compared to 5% polyvidone iodine–alcohol [346]. The catheter tip is also a major source of contamination. For this, its disinfection with appropriate antiseptic or antimicrobial ointments is recommended. Use of a povidone–iodine antiseptic ointment or bacitracin/gramicidin/polymyxin ointment at the exit site after catheter insertion is recommended [335]. The use of sterile gloves, a sterile long-sleeved gown, mask, and large sterile sheath sheet during insertion of a CVC are essential for the prevention of CA-BSIs. A checklist should also be used to improve adherence to procedures at the time of insertion [347]. After insertion of the catheter, the risk of infection should decrease with the use of aseptic techniques. However, insertion and maintenance of VCs by inexperienced personnel could increase the risk of catheter colonization and the development of infection. Having an experienced infusion therapy team in place to insert and maintain catheters decreases CA-BSI levels up to eight times [27].

### 7.3. Catheter Insertion Site

The catheter insertion site is an important parameter whose choice should be based on both the benefits and risks of the procedure (infection, thrombosis, and mechanical complications). The subclavian site is the ideal insertion site for CVCs, which helps reduce infectious complications [347]. This is probably explained by the fact that the subclavian route has the longest subcutaneous distance between the skin and the entrance to the vessel [348]. In addition, according to previous studies, subclavian catheterization was associated with a lower risk of infectious and thrombotic complications than femoral and jugular catheterization [349,350,351].

### 7.4. Catheter Lock Solutions

Another approach that shows promise for the prevention of CA-BSIs is antimicrobial lock therapy. It involves instilling a highly concentrated antimicrobial solution into the lumen of the catheter when not in use [352,353] to remove the blood so that the occlusion and bacterial growth are minimized [5] and also preventing biofilm formation [354]. This technique is useful especially in cases of uncomplicated long-term CA-BSIs caused by pathogens [47]. A variety of antimicrobial agents can be used such as heparin (anti-occlusion) [355,356], vancomycin, gentamicin (antibiotics) [357,358], citrate, ethanol, and taurolidine (antimicrobials) [357,359,360,361]. Antibiotics are generally used for therapeutic measures once a CA-BSI has been diagnosed, while heparin, citrate, ethanol, and taurolidine are used prophylactically [5]. Furthermore, Kumar et al. [362] demonstrated that using S-nitroso-N-acetyl-l-cysteine ethyl ester (SNACET)is very effective; this is able to generate nitric oxide with antimicrobial properties as a catheter locking solution. Indeed, a significant reduction of 99% in the adhesion of *S. aureus* and *E. coli* on catheters was observed.

### 7.5. Dressing

To prevent complications for patients, a dressing is often placed where the integrity of the skin is compromised. Several materials are used as dressing for VCs [362,363]. A gauze dressing is often used when blood seeps from the catheter insertion site. However, their use increases the risk of bacterial contamination and infection [347,364]. Transparent semi-permeable dressings are widely used and allow continuous observation of the skin insertion site and reduce the risk of extrinsic colonization. They should be changed immediately if they become wet, loose, or soiled [347]. The risk of CA-BSI increases more than 3-fold after rupture of the second dressing and more than 12-fold if the final dressing is ruptured [365,366]. The chlorhexidine-impregnated dressing, an innovative strategy, shows promising results in the prevention of infections linked to CVCs, with a reduction in colonization (6.5% versus 13.2%) [367] and infection (1.51/1000 versus 5.87/1000 catheter-days) compared to traditional dressings [368]. Moreover, Puig-Asensio et al. [369] reported that chlorhexidine dressings reduced the risk of CA-BSIs in patients with short-term CVCs, including those with an onco-hematological disease. There is also other evidence that shows that dressings impregnated with chlorhexidine may reduce the risk of CVC-BSI, compared to standard polyurethane dressings, and other types of non-impregnated dressings (gauze and tape dressing) [369,370,371,372,373]. A recent study conducted by Hou et al. [374] found that the chlorhexidine gluconate gel dressings used more effectively reduced the risk of CVC-BSI in patients unlike the chlorhexidine gluconate sponge dressings.

### 7.6. Antimicrobial Agents Release

Several strategies using coating or impregnating catheters with antibiotics/antimicrobials, peptides, metals, nitric oxide, or other compounds have been developed to prevent biofilm formation and constitute a promising alternative to reduce infection rates and CA-BSIs [375,376]. The use of antimicrobial agents as a coating is the most popular approach due to their ability to target microorganisms in different ways [377]. Inhibition of bacterial adhesion on catheter surfaces could be prevented by releasing the antimicrobial agent [378]. This approach aims to attach the antimicrobial agent to the catheters by adsorption, which will diffuse after exposure to body fluids [379]. This approach allows the release of high doses of the antimicrobial agent without exceeding the toxic threshold, reducing the development of resistance. However, the drawback with this technique is that the release is uncontrolled and lacks long-term properties [380,381].

Catheters coated with chlorhexidine–silver sulfadiazine, minocycline–rifampicin and miconazole and rifampin are the most commonly studied and are associated with a decreased prevalence of catheter colonization and CA-BSIs [382,383,384,385]. In fact, it has been reported that these impregnated catheters had the potential to reduce the risk of colonization of these devices and the incidence rates of CA-BSIs per 1000 catheter days [5]. Among catheters based on antimicrobial agent release which have been approved and commercialized, there is ARROWg+ard^®^ (chlorhexidine and silver sulfadiazine coating), Spectrum^®^ (minocycline and rifampin coating) and Chlorag+ard^®^ (chlorhexidine coating) [5]. Other agents are able to reduce bacterial colonization on venous catheter surfaces. Table 2 summarizes most research studies which tested the antimicrobial agent coating/imprenated and surface modifications approaches for the prevention of bacterial colonization and CA-BSI.

### 7.7. Contact Kill Systems

Contact destruction of bacteria relies on the use of antimicrobials grafted onto the surface of catheters to form a lethal barrier for these pathogens [5]. Indeed, these antimicrobial molecules are mainly cationic or enzymes that bind covalently to the surface of the catheter via hydrophobic polymer chains, and kill these bacteria on contact via membrane interactions.Additionally, this strategy exhibits longer antimicrobial activity and low toxicity [379] and does not output biocides in body fluids [397]. Several compounds such as quaternary ammonium compounds, peptides, graphene derivated (Table 2) have been evaluated as promising contact killing agents. However, the major concern with this strategy is that the bioactive surface can be inactivated when coated with proteins from body fluids [379]. For that, further research studies are needed to improve the strategy.

### 7.8. Antifouling Approaches

**Figure 2:**
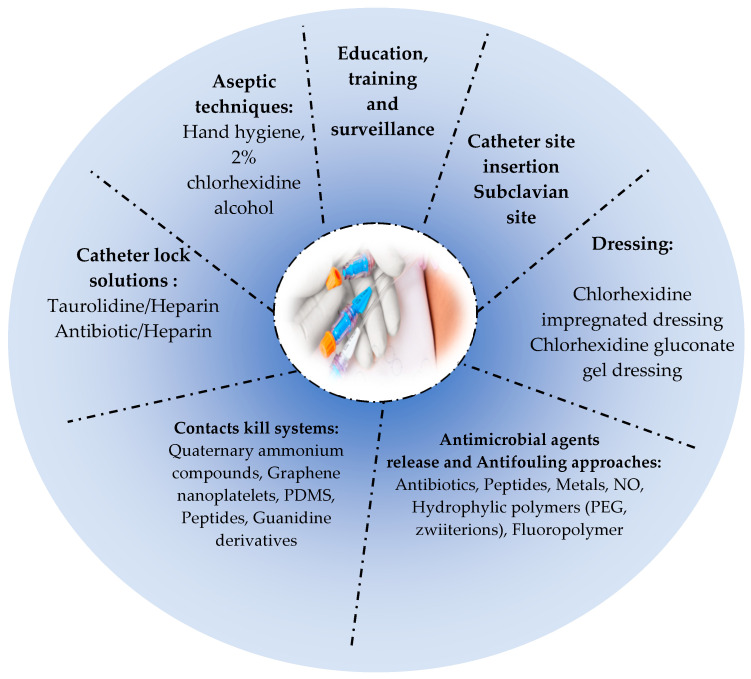
Main prevention strategies of CA-BSIs.

Surface modifications, such as hydrophilic polymeric surface coatings, work also by reducing microbial adhesion to the catheter surface, thereby minimizing infection [375]. Indeed, surface hydration is an important parameter of antifouling coatings due to the water layer formed on the surface of the polymer, which acts as a barrier preventing bacterial adhesion and proteins adsorption [376].The most hydrophilic polymers used in the antifouling approach are poly(ethylene glycol) (PEG) (most commonly used), poly-2-hydroxyethyl methacrylate, poly(2-hydroxypropyl acrylamide), dextran, and zwitterionic polymers [400]. The immobilization of zwitterionic compounds or PEG provides promising results for CR-BSI prevention [398,401]. A recent technology which is the fluoro-passivation of catheters has emerged as an effective approach which consists of coating the catheter with fluoropolymer to increase its biocompatibility and reduce infection [337]. Among the coated catheters commercialized are the AngioDynamics BioFlo PICC catheter (endexo) and the CerebroFlo extraventricular drain catheter (endexo) [337]. Furthermore, another prevention way is the use of materials characterized by low energy, such as hydrophobic polymers (PTFE) [5].A few studies on surface modifications of venous catheters are cited in Table 2. Figure 2 illustrates the main prevention strategies of CA-BSIs.

## 8. Prevention of CA-UTIs

CA-UTI is one of the most common device-related infections in which preventive measures should be taken [402]. These precautions to prevent the transmission of MDR-bacteria must be scrupulously observed in catheterized patients and also limit the uncontrolled use of antibiotics [403].

### 8.1. Avoidance of Urinary Catheter Use

The main CA-UTI prevention strategy is to avoid or reduce the use of catheters. Overall, UCs are overused and placed for inappropriate indications in 21–50% of catheterized patients [403]. Accepted indications for the use of a catheter are considered the first step in limiting their uses. Among these limitations are the following: urological surgery, monitoring of urine flow in seriously ill patients, management of acute urinary retention and urinary obstruction, or for end-of-life care to improve patient comfort [404]. Limiting the duration of catheterization is also very important. Indeed, when a catheter is placed, it must be removed quickly once it is no longer needed [404]. Healthcare providers should be aware of the existence of the UC. Therefore, catheter remainder interventions that include a verbal/written reminder, a sticker reminder on the patient’s chart or an electronic reminder that indicates that a urinary catheter is still in place is a good prevention strategy. Another type of intervention called a “stop order” that requires the clinician (nurse or doctor) to remove the catheter after a period of catheterization or a condition has occurred, unless the catheter remains clinically appropriate, can be followed also in case the reminders are ignored [405]. Institutional policies should also reduce the use of peri-operative catheters by encouraging early removal of post-operative catheters and monitoring bladder volume using ultrasound bladder scanners [404].

### 8.2. Alternatives to Indwelling UC

Studies have shown decreased UTIs or deaths in patients who used condom catheters. In addition, this type of catheter seems to be less painful and more comfortable than indwelling catheters. Therefore, condom catheters may be an alternative for patients with retained or obstructed bladder. It has also been reported that the use of intermittent catheterization may be beneficial in long-term catheterized patients with neurogenic bladder or after hip surgery has reduced the risk of bacteriuria thereby minimizing the need for an indwelling catheter [403].

### 8.3. Education and Training

Health care personnel and others who handle urinary catheters should be trained in the procedures for inserting, maintaining, and removing urinary catheters. Education should also be offered on catheter associated urinary tract infections, complications of urinary catheterization, and alternatives to indwelling catheters [406].

### 8.4. Aseptic Techniques for Insertion and Maintenance of UCs

When indwelling catheterization is required, aseptic catheter insertion and maintenance are recommended to prevent CA-UTIs. For this, UCs must be placed by a qualified healthcare professional [403]. Among these recommendations are the following: hand washing with soap and water should be carried out immediately before and after handling a urinary catheter; the surface of the urethral meatus must also be clean before insertion of the urinary catheter; the catheter must be attached to the patient’s thigh to avoid lesions of the urethral meatus; in case of any skin irritation, the catheter should be changed immediately; sterile and closed urine drainage should be used to reduce the risk of infections; finally, irrigation of the bladder with normal saline or a solution containing antibiotics is not recommended, except in cases of obstruction [407].

### 8.5. Antimicrobial Coatings

Although improved hygiene procedures, replacement of UCs, and the use of prophylactic antibiotics have helped to reduce the incidence of CA-UTIs, it has not been avoided sufficiently. One of the most promising approaches is the use of antimicrobial coatings on UC surfaces to prevent CA-UTIs but more specifically to prevent adhesion, biofilm formation and encrustation of catheters [32,408,409,410,411]. Such strategies reduce the viability of pathogens by inhibiting the metabolic pathways necessary for their survival such as inhibiting the synthesis of nucleic acids and proteins involved in cell wall synthesis [410]. However, the development of these devices with antimicrobial surfaces must meet certain requirements, including easy and reproducible production, resistance to mechanical stresses, biocompatible and non-toxic, antimicrobial efficacy for a long time, and finally avoiding the development of resistance [411]. Among the antimicrobial agents used for UC coating are the following: metal (silver, nanoparticles), antibiotics, nitric oxide, antimicrobial peptides, bacteriophages [410]. Table 3 summarizes most research studies which tested the antimicrobial agent coating for the prevention of bacterial colonization and CA-UTIs.

Metals or composite nanoparticles represent suitable alternatives for CA-UTI prevention and biofilm-related infections [434]. Several studies [409,435,436,437,438,439] have reported that UCs coated with thin-films of silver alloy could reduce bacterial adhesion but also the incidence of asymptomatic bacteriuria and CA-UTI. Other metals have been tested as coatings such as copper (Cu) [412].

Recently, novel advance, namely nanoparticles, constitutes a promising approach in biomedical devices [47]. Several studies reported the efficiency of a silver nanoparticle coating method in colonization prevention of several pathogens such as *E. coli*, *P. mirabilis*, *P. aeruginosa*, *Stapholococcus* spp., and *Enterococcus* spp. [413,429,440,441,442,443]. Throughout the years, other nanoparticles have been studied as coating, including green–silver nanoparticles [444,445], gold nanoparticles [446], copper nanoparticles [447] and zinc-doped(Zn) copper oxide (CuO) nanoparticles [414,448].

Antibiotics have been extensively studied over the years and despite the emergence of MDR-pathogenic bacteria, several studies have shown the effectiveness of many antibiotics on infections caused by Gram-negative and Gram-positive bacteria [449]. Moreover, antibiotics are often used for CA-UTI treatment [378]. However, due to their high cost and conflicting results between in vitro studies and clinical trials, their use is questionable [377].Antibiotics that have been commonly studied are such as nitrofurazone. Nitrofurazone-coated UCs was tested against several pathogen biofilms (*E. coli*, *P. aeruginosa*, *S. epidermidis*, *En. faecalis*) with promising results [450,451,452]. However, their carcinogenic potential in animal models induced their removed from the market and prohibition by the FDA [410]. Other antibiotics such as gentamicin [453], chlorhexidine [454], ciprofloxacin [455], norfloxacin [456], triclosan [457], and sparfloxacin [418] have been tested as a coating agent. Although giving promising results, the antibiotic-based approach favors the apparition of bacterial resistance for long-term catheters (e.g., triclosan), leading to more serious infections [434,458].

For that, the antimicrobial peptides (AMPs) are considered as the most promising strategies to conventional antibiotics [420].AMPs are host defense peptides widely used in the treatment of biofilms associated with several clinical pathogens and kill them by membrane permeabilization [459,460]. Recently, these peptides (e.g., RK1, RK2, CWR11, Bmap-28, E6, Chain 201D) were tested as coating agents for UCs to prevent biofilm formation and CA-UTIs [419,461,462,463].

In other studies, nitric oxide (NO), a natural gas molecule, with a short half-life, has been demonstrated as being able to protect the host against several pathogens [464]. Its mechanism of action is that it binds covalently to DNA, proteins, and lipids to inhibit or kill the pathogen [377]. The approach based on NO appears to be a promising alternative to combat bacterial infections and the formation of biofilms [465,466].

Bacteriphages have also been suggested as a new strategy to combat bacterial biofilms. They specifically infect bacteria and disrupt their metabolism to self-replicate and then, kill them [128,467]. In addition to their specificity and self replication as advantages, they are able to degrade biofilm matrix and prevent resistance development, while the treatment is improved when phages cocktail is used [378,410,468]. Phage therapy has been used to treat wide bacterial infections with little or no side effects constituting a promising technology in clinical application [469]. Until now, several phage-based coating UCs have been developed and tested for uropathogens including *E. coli*, *K. pneumoniae*, *P. mirabilis*, *P. aeruginosa*, and *Staphylococcus* spp. [468,470,471,472,473].

### 8.6. Antifouling Approaches

Antifouling approaches can also prevent bacterial adhesion on UCs and biofilm formation by repelling them without harming them [377]. The principle of these strategies is the acquisition of anti-adhesive properties by physicochemical modifications of catheter surfaces in order to prevent bacterial adhesion in addition to a good antibacterial activity and low toxicity [376,434]. Moreover, this approach provides the advantage of low risk of drug resistance emergence [474]. Additionally, the hydration layer increases patient comfort due to low friction during UC placement [434]. There are wide antifouling approaches, especially hydrogels which are the most popular due to their hydrophilic structure which reduces bacterial growth [475]. Poly(tetrafluoroethylene) (PTFE), poly(ethylene glycol) (PEG), polyzwitterion, and enzyme coating were also studied [377] to prevent the development of biofilms on UC surfaces and CA-UTI prevention. These polymers repel foulants due to the formation of a hydration layer on the surface [449]. Among the hydrophilic coatings, some of them have been already commercialized including HydroPlus™ from Boston Scientific, AQ^®^ from Cook Urological, heparin-based coating Endo-Sof™ Radiance™ from Cook Urological, and SL-6 from Applied Medical [434].

Hydrogels are hydrophilic polymers widely studied as coatings due to their excellent hydropilicity, high hydration, and porous structures [476]. Several studies reported the efficiency of this approach [477,478,479]. However, it was reported that this approach caused the encrustation of catheters which is contradictory to other results [480]. Further studies are needed to provide more information about long-term prevention of CA-UTIs and biofilm formation and validate their effectiveness.

Another polymer used for coating is poly(tetrafluoroethylene) (PTFE), called also teflon which is characterized by high non-stick properties and resistance to bacterial adhesion, making it an excellent option for biofilm prevention [378,409]. The teflon-coated catheters are commercially available from Bard Medical [377]. Various early studies have been also conducted for the same purpose [429,481,482].

Similary, polyethylene glycol (PEG) possesses nonimmunogenic, nonantigenic, and protein repellent properties thus appearing to be a good antifouling agent [377].

Polyzwitterions that contain both cationic and anionic ions constitute also promising antifouling agents for the coating of UCs due to their superhydrophilicity [483]. Researchers designed different polyzwitterion silicone catheter surfaces and studied their effectiveness including sulfobetaine methacrylate (SBMA) [428], copolymer-coated Ti6Al4V (Ti6Al4V@DMA-MPC) [484], polysulfobetaine (PSB) [485], poly(sulfobetaine methacrylate) (pSBMA), and poly(carboxybetaine methacrylate) (pCBMA) [486].

**Figure 3:**
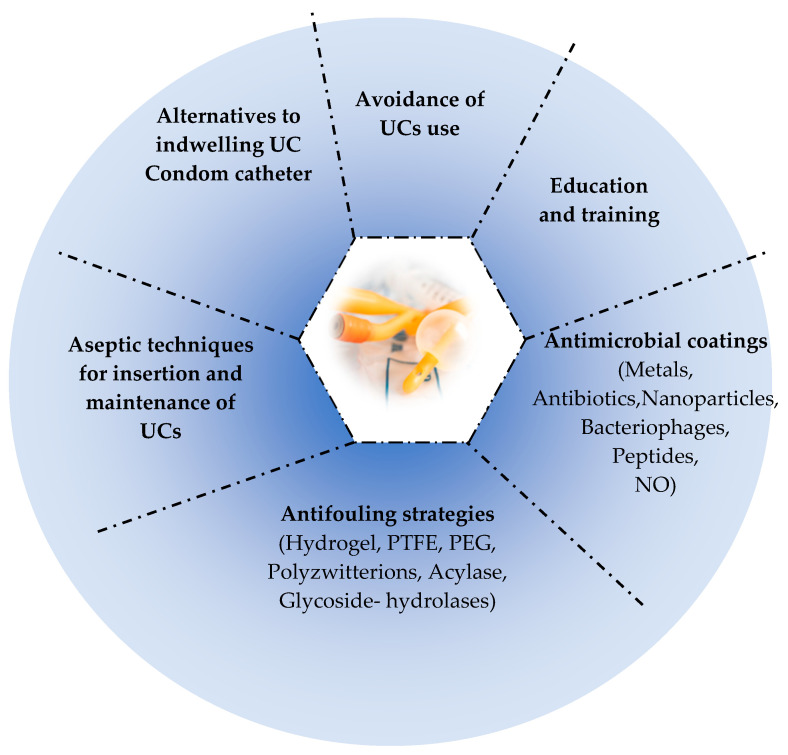
Main prevention strategies of CA-UTIs.

In the recent past, the effectiveness of the enzymes was evaluated toward bacterial adhesion [377]. Furthermore, the enzymes are natural, safe, and non-toxic to other than their target cells which is an advantage. They were recently studied in the UC-coatings field [449,487]. Among the enzymes already tested are the following: acylase, cellobiase dehydrogenase, α-chymotrypsin, and glycoside hydrolases [377]. Table 3 summarizes most of the studies showing the effectiveness of different antifouling approaches. Figure 3 illustrates the main prevention strategies of CA-UTIs.

## 9. Conclusions

Medical device-associated biofilm infections, mainly catheter associated bloodstream infections and catheter associated urinary tract infections, which are the most common infections in healthcare, constitute a real problem in hospitals. In addition to the global emergence of multidrug resistance, the biofilm formation on these devices, especially the presence of persistent cells, makes these infections worse, causing the recalcitrance of infections and therapeutic failure, thereby increasing the rate of morbidity, mortality, healthcare cost, and length of hospitalization.

However, the risk of these infections could be reduced by respecting the prevention guidelines, including educating healthcare personnel, hygiene, limiting use, choice of catheter insertion site, and the antimicrobial lock therapy. In recent years, novel and effective advanced strategies have been developed as contact kill systems—antimicrobial-coated catheters with metals, nanoparticles, phages, antibiotics, antimicrobial peptides and other compounds—and have helped to reduce bacterial adhesion, biofilm formation, catheter encrustation, cytotoxicity, and complications for patients. Antifouling approaches also constitute promising alternatives to prevent medical device associated infections. Despite all the in vitro and in vivo studies that have been conducted in this area, no ideal strategy has been found until now due to the divergence of the results obtained. Further, additional research studies, notably clinical trials, are still needed to develop biocompatible strategies and fully validate their efficiency in order to prevent and fight medical device-associated biofilm infections, especially for long-term catheters.

## Figures and Tables

**Table 1:** Main medical device-associated bacteria and their virulence factors.

Pathogen	Virulence Factor	Characteristics and Function	Reference
*E. coli*	Type I *fimbriae*	Encoded by *fim* operon located on the chromosome of UPEC isolates; Binding specifically to D-mannose which is found on the glycoproteins of the epithelial cells; Represents 95% of the virulence factors of *E. coli*; A major adhesin in colonization of UCs and biofilm formation during CA-UTIs.	[94,97,98,103,104,105]
	P *fimbriae*	Pyelonephritis-associated pili (Pap); The second important adhesin expressed by UPEC and coded by the *pap* operon; Binding to di-galactoside moiety present in the urinary tract epithelium.	[100,106,107]
	Curli Csg A	Key components of the extracellular biofilm matrix of *E. coli* in which CsgA is the major subunit of curli; Bacterial binding with fibronectin, laminin, plasminogen; Aggregation, adhesion to surfaces, and biofilm development.	[108,109]
	PGA (Poly-β-1,6-N-acetyl-D-glucosamine)	Encoded by *pgaABCD*operon; A primary component of the biofilm matrix; Attachment of *E. coli* to surfaces and autoagregation of cells.	[82,89]
	Ag 43 (Antigen 43 adhesin)	One of the major auto-transporter in *E. coli* encoded by the gene *flu*; Translocation to the outer membrane; Adhesion and auto-aggregation (cell-to-cell) facilitating the formation of the biofilm.	[89]
	Hemolysin F	Encoded by the gene *hlyF*; Over-expression of *hlyF* promotes the biosynthesis of the outer membrane vesicles (OMVs) which release toxins involved in virulence.	[110,111]
	α-hemolysin	Encoded by the gene *hlyA*; A pore-forming cytotoxin, responsible for lysis of the cell membrane of hosts (leukocytes, erythrocytes, and endothelial cells).	[112]
	Siderophores	Survival and colonization in iron-deficient sites.	[78]
	CNF-1 (cytotoxic necrotizing factor 1)	Responsible for the apoptosis of urothelial cells then increase bacterial entry to the bladder.	[89]
	LPS (Lipopolysacharide)	An endotoxin that induces septic shock caused by over-expression of pro-inflammatory cytokines.	[113]
	Capsule	Adherence to host cells; Biofilm formation; Binding to C4 binding protein (C4BP) and inhibits complement cascade; Bacterial protection from phagocytosis; Binding to catioinc antimicrobial agents; Bacterial protection from macrophage recognition.	[114]
	*Quorum sensing*	Main *quorum sensing* systems: LuxS and SdiA, producers of autoinducer-2 molecules; Cell-to-cell communication; Role in bacterial behaviour coordination and regulation of virulence genes.	[115]
*K. pneumoniae*	Types 1 *fimbriae*	Encoded by *fimAICDFGHK* operon; Adhesion mediation to mannose-containing structures present on host tissue and extracellular matrix; A major role in biofilm formation on UCs, invasion and colonization of host cells, and persistence in catheters-associated infections.	[116,117]
	Types 3 *fimbriae*	Encoded by *mrkABCD* operon; Adhesion to different structures in kidney, lung tissue, endothelial and bladder epithelial cells; A major role in biofilm formation on UCs, invasion and colonization of host cells, and persistence in catheter-associated infections.	
	PGA (poly-β-1,6-N-acetyl-D-glucosamine)	Encoded by *pgaABCD* operon; Cell-cell communication; Intercellular adhesion; Adhesion to abiotic surfaces.	[118]
	Capsule polysaccharides (*magA*, *k2A* and *wcaG)*	Resistance to phagocytosis; Complement-mediated lysis inhibition and opsonization; Host defense escape.	[119,120]
	Lipopolysaccharides (*wabG*, *uge*, *ycfM)*	Inhibition of complement pathway; Inactivation of the seditious response; Block the effect of peptides via lipid A; Host defence escape.	
	Siderophores (*iutA*, *iroN*, *entB)*	Acquisition of iron from host iron-chelating proteins for survival and growth during infections; Biofilm formation; Host defence escape.	
	*Quorum sensing*	*QS* regulator systems: Type 2 *quorum sensing luxS*; Cell–cell communication; Intercellular adhesion and adhesion to abiotic surfaces; Bacterial behaviour coordination; Regulation of virulence genes.	[118,121]
*P. mirabilis*	MR/P (Mannose resistant *Proteus*-like *fimbriae*)	Binding to uroepithelial cells; Attachment to urinary catheters; Biofilm formation.	[33,122,123]
	PMP (*P. mirabilis* P-like pili)		
	PMF (*P. mirabilisfimbriae*)		
	ATF (Ambient-temperature *fimbriae*)		
	UCA (Uroepithelial cell adhesin)		
	Flagella	A major role in swarming; Migration of pathogenic strains to the upper urinary tract, causing pyelonephritis; Dispersion of biofilm from urinary catheters to the urinary tract.	[33,124,125]
	Hemolysin *HpmA*	Ability to lyse erythrocytes, bladder epithelial cells, and monocytes; High cytotoxicity towards human renal proximal tubular epithelial cells (HRPTECs); Dessimination of *P. mirabilis* into the kidneys and development of pyelonephritis.	[122,126,127]
	*Proteus* toxigenin *Pta*	An autotransporter which promotes autoaggregation of the bacteria. Mediates lysis of bladder epithelial cells.	
	*ZaPA*(zinc metalloproteinases)	Degradation of immunoglobulins IgA and IgG, human β-defensin 1, and other celluar components (fibronectin, collagen); Escape immune responses during infection.	
	LPS (Lipopolysccharides)	Mediation of the induction of proinflammatory cytokine responses; Induction of apoptosis; Septic shock.	
	Iron acquisition system	Production of iron carriers to take iron from the host and use it for its survival during urinary infections.	
	Urease (a nickel-dependent metalloenzyme)	Degradation of urea into carbon dioxide and ammonia, increasing the urine pH up to 8.2; Formation of crystals, struvite (ammonium and magnesium phosphate) and apatite (calcium phosphate); Causing the obstruction of urinary catheters; Causing pyelonephritis and increasing the risk of sepsis.	[128,129]
	*Quorum sensing*	*QS* system regulator: luxS/luxR system; Autoinducer-1 molecules controlled by the *luxR* genes and autoinducer-2 molecules controlled by the *luxS* genes; Role in swarmingcoordination; Regulation of biofilm formation and virulence genes expression.	[125]
*P. aeruginosa*	Flagella Type IV pili	Important role swimming, twitching, and swarming motility; Adhesion to host epithelial cells; Attachment to surfaces and biofilm formation.	[130]
	LPS (Lipopolysccharides)	Antibiotic tolerance, tissue damage, and biofilm formation; The complement system induction; Activation of inflammatory cytokines TNF-α and IL-1β; Induction of immune responses via Toll-like receptor 4 (TLR4) and cystic fibrosis transmembrane conductance regulator (CFTR); Induction of phagocytosis; Neutrophil activation for neutrophil extracellular trap (NET) releasing which contain pathogens.	[131,132]
	Exopolysaccharides (alginate, PEL and PSL)	Crucial role in initial attachment to surface; Biofilm formation, its stability and maintenance; Bacterial protection from phagocytosis and opsonization, Biofilm maturation, and prevention of antibiotic diffusion.	[133]
	OMVs (Outer membrane vesicles)	Expression of 26 OMPs of which the porin OprF is the most abundant; Transport of molecules (e.g.,toluene, siderophores, nitrates, and nitrites); Bacterial adhesion and biofilm formation; Implication in drug resistance; Protection from macrophage clearance during chronic infections; Remove of competing bacteria from the environment during infections.	[134,135]
	Siderophores (Pyoverdine and Pyochelin)	Iron chelation from transferrin and lactoferrin needed during growth and virulence.	[131,136]
	Elastase A [LasA] and B [LasB]	Degradation of elastin; Host tissues damage.	
	Protease IV	Degradation ofcomplement components, immunoglobulins, and surfactant protein; Raising of bacterial infection via fibrinogen, lactoferrin, transferrin degradation; Host tissues damage.	
	toxins (Pyocyanin; T3SS effectors [ExoS, ExoT, ExoU and ExoY]; Exolysin [ExlA]; Exotoxin A [PEA]; Lipase A [LipA] and Leukocidin)	Pyocyanin: induction of oxidative stress for the host to avoid bacterial elimination; T3SS effectors: inhibition of phagocytosis and bacterial elimination, disruption of the host actin cytoskeleton, and apoptosis induction; ExlA: induction of membrane permeabilization and cell death through its cytolysin activity; PEA: inhibition of host protein synthesis by ADP ribosylation activity and stimulatation of programmed cell death; Lipase A: degradation of lipid dipalmitoylphosphatidylcholine (lung surfactant) and drug resistance mediation by interacting with alginate; Leukocidin: leukocytes swelling by increased permeability of their membrane.	
	*Quorum sensing*	Main*QS*systems: *Las*, *Rhl*, *Pqs* and *Iqs*; Regulationof biofilm formation and other virulence factors; Coordination of bacterial behaviour and persistance during infection.	[137,138]
*A. baumannii*	Csu (Chaperone-usher pili)	Encoded by *csuA/BABCDE* operon; Involved in the initial ahesion onto abiotic surfaces but not biotic surfaces.	[139,140]
	OmpA (Outer membrane protein A)	A β-barrel porin, one of the most abundant porins in the outer membrane of *A. baumannii.* Role in the virulence of *A. baumannii*, including interaction with the host, cytotoxicity, apoptosis, and biofilm formation.	[139,141,142,143]
	Bap (Biofilm-associated protein)	Formation of water channels; Maitainingthe structure and integrety of biofilm; Biofilm formation on abiotic and biotic surfaces.	[139,144]
	PNAG (Poly-N-acetyl β-1-6 glucosamine)	Major component of the *A. baumannii* biofilm matrix and encoded by the *pgaABCD* operon; Role in the integrity of the biofilm; Tolerance to desiccation stress; Incredible persistence in natural environments and care facilities.	[144,145]
	Type V secretion systems	Transport exoproteins; Transport mobile genetic elements; Role in bacterial competition; Biofilm formation on abiotic and biotic surfaces.	
	Phospholipases C and D	Hydrolytic activity towards phosphatidylcholine; Hemolytic activity against erythrocytes.	
	Capsule	A protection barrier. Resistance to some antibiotics. Regulation of the *K locus* genes for exopolysaccharides production, important for biofilm formation.	
	Iron-chelator proteins	Uptake of iron from host environnement in iron deficiency conditions.	[140]
	*Quorum sensing*	Two-component regulatory system: AbaI/AbaR system Regulation of several virulence factors such as biofilm formation and motility.	[139]
*S. aureus*	PIA (Intracellular adhesion polysaccharide) or PNAG (o poly-N-acetyl β-1-6 glucosamine).	Encoded by the *icaADBC* operon; Important in cell-to-cell adhesion, adhesion to surfaces, biofilm formation; Antimicrobial resistance; Immune evasion; Bacterial protection from phagocytosis.	[146,147,148]
	FnBPA and FnBPB (Fibronectin-binding proteins)	Categorised as “microbial surface component recognising adhesive matrix molecules (MSCRAMM)”; Implication in binding host matrix components (fibronectin, fibrinogen, collagen, elastin, laminin); Initial cell attachment and/or biofilm formation; Implication in colonization; Immune evasion.	[146,149,150]
	ClfA and ClfB (Clumping factors)		
	Can (Collagen adhesin)		
	EbpS (Elastin binding protein)		
	Fib (Fibrinogen binding protein)		
	Eno (Laminin-binding protein)		
	SdrC, SdrD, SdrE (Serine aspartate repeat proteins C, D, and E)		
	Atl (Autolysin)	Primary attachment through non-specific hydrophobic interactions with uncoated surfaces; Bindin to host extracellular matrix proteins and involvment in cell separation during cell division.	[151,152]
	Bap (Biofilm-associated protein)	Contributionin initial adhesion to abiotic surfaces; Induction of strong intercellular adhesion.	[153,154]
	*Quorum sensing*	Global regulatory systems [accessory gene regulator (*agr*), staphylococcal accessory element (*sae*), the staphylococcal accessory regulator A (*sarA*)]; Regulation of the expression of virulence factor secretion and biofilm formation.	[155]
*S. epidermidis*	PIA (Intracellular adhesion polysaccharide)	Adhesion and biofilm accumulation.	[156]
	AtlE (Autolysin E)	Attachment to plastic surfaces.	[157]
	Bap homolog protein Bhp	Adherence to a polystyrene surface, intercellular adhesion, and biofilm formation.	
	Ssp-1,Ssp-2 (Staphylococcal surface proteins 1 and 2)	Cell-to-cell adhesion; Biofilm formation.	
	Serine-aspartate repeat protein G (SdrG/Fbe) binding to fibrinogen	Adhesion to the host proteins (fibrinogen, collagen, fibronectin); Adhesion to abiotic surfaces.	[158]
	SdrF (Serine-aspartate repeat protein F) binding to collagen		
	Extracellular matrix-binding protein (Embp)		
	Phenol-soluble modulins (PSMα, PSMδ, PSMε, δ-toxin [PSMγ], and PSMβ [PSMβ1, PSMβ2])	Acquisition of the characteristic three-dimensional structure-like mushrooms; Role in biofilmdispersion.	[157,159]
	Homolog of the SspB	Role in the degradation/dispersion of the biofilm.	[157]
	Homolog of SspA V8		
	Metalloprotease SepA		
	Nucleases		
	*Quorum sensing*	Two key systems: the *agr* and the *sar* regulators; Expression/repression of virulence genes in a coordinated manner during infection.	[160]
*En. faecalis*	Esp (Enterococcal surface protein)	Primary adhesion in UTIs; Colonization of the urinary tract.	[161,162]
	Asa1 (Aggregation substance)	Adhesion to host cells and bacterial aggregation.	
	Collagen binding protein (Ace)	Adhesion to extracellular matrix and type 1 collagen.	
	EfaA(*En. faecalis* endocarditis antigen A)	Adhesion to biotic and abiotic surfaces.	
	Epa (Enterococcal polysaccharide antigen)	Colonization, translocation through epithelial cells, bacterial adhesion, biofilm formation, and antibiotic resistance.	[163]
	Ebp A,B,C (Endocarditis and Biofilm-Associated Pili)	Important in initial attachment, biofilm formation, and endocarditis.	[164,165]
	CylA (Cytolysin A)	Killing other bacteria (especially Gram-negative bacteria) and eukaryotic cells (red blood cells); Biofilm formation.	[162,165,166]
	Hyl (Hyaluronidase)	Degradation of hyaluronic acid to permeabilize host tissues; Induction of autoimmune diseases.	
	GelE (Gelatinase)	Degradation of the collagen adhesion protein (Ace) which contributes in colonization and biofilm formation; Degradation of gelatin, collagen, fibrin, fibrinogen, hemoglobin, and complement components (C3, C3a, C5a); Cell lysis.	
	SprE (Serine protease)	Degradation of casein; Release of eDNA.	
	*Quorum sensing*	Regulator system: the Fsr (fecal streptococci regulator) *quorum-sensing* system, encoded by *fsrA*, *fsrB* and *fsrC* genes; Regulation of communication through peptide pheromones cpd, cob, and ccf; Control of biofilm formation via regulation of gelatinase production.	[167]

**Table 2:** The effectiveness of different antimicrobial agent coating and surface modification approaches.

Strategy	Agent Used	Approach Used for Coating VC	Microorganism	Reference
Release killing	*Antirhumatic*			
	Auranofin	Auranofin-coated polyurethane catheter.	MRSA	[386]
Release killing	Auranofin	Auranofin-coated polyurethane catheter.	*S. aureus*	[387]
Release killing	*Guanidine derivated*			
	poly(hexamethylene biguanide) hydrochloride–sodium stearate (PHMB–SS)	Coating developed using electrostatic interaction based on polyelectrolyte.	*E. coli* *S. aureus*	[388]
	*Antimicrobial peptides*			
Contact killing	ε-Poly-ʟ-lysine	Electrostatic interaction between cation PL and anion surfactant, 1,4-bis(2-ethylhexyl) sodium sulfosuccinate (AOT).	*E. coli* *S. aureus*	[389]
Contact killing	Poly(methacrylic acid) (PMAA)	Polyurethane surface-initiated atom-transfer radical polymerization (SI-ATRP)	*E. coli* *S. aureus*	[390]
	*Nitrix oxide*			
Release killing	Boron carbon nitride (BCN)	Boron carbon nitride nano-coating using RF magnetron sputtering technique.	*E. coli*	[391]
Release killing	*S*-nitroso-*N*-acetyl-penicillamine (SNAP)	Incorporation of a nitric oxide (NO) donor molecule, *S*-nitroso-*N*-acetyl-penicillamine (SNAP) in a hydrophobic medical grade polymer, Elasteon-E2As and coated with fibronogen.	*E. coli* *S. aureus*	[392]
	*Metal*			
Release killing	Silver nanoparticles (AgNP) Zinc oxide (ZnO)	Incorporation of silver nanoparticles (AgNP) and ZnO nanowires with polyvinylchloride(PVC).	*S. aureus*	[393]
Release killing	Silver	Synthesized novel silver(I) cyanoximates Ag(ACO), Ag(BCO), Ag(CCO), Ag(ECO), Ag(PiCO), Ag(PICO) (yellow and red polymorphs), Ag(BIHCO), Ag(BIMCO), Ag(BOCO), Ag(BTCO), Ag(MCO) and Ag(PiPCO).	*P. aeruginosa* *S. aureus*	[394]
	*Quaternary ammonium compounds*			
Contact killing	Quaternary ammonium thiol compound (Q8-SH)	Grafting a quaternary ammonium thiol compound (Q8-SH) to a thermoplastic polyurethane containing allyl ether (allyl-TPU) side-chain functionality.	*E. coli* *P. aeruginosa* *S. aureus*	[395]
	Graphene derivated			
Contact killing	Graphene oxide	Immobilization of oxidized graphene nanoplatelets (GNP-M5ox) on the surface of silicone rubber by dip and spray coating.	*S. epidermidis*	[396]
	*Other compounds*			
Contact killing	poly(dimethylsiloxane) (PDMS)	Hydrophobic hyperbranched coating resin was covalently attached to PDMS.	*E. coli* *P. mirabilis* *S. aureus* *S. epidermidis*	[397]
	*Hydrophilic polymer*			
Surface modification	Poly(ethylene glycol) PEG	Microcrystalline sulfamethoxazole (SMZ) and trimethoprim (TMP) were immobilized with PEG.	*E. coli* *S. aureus*	[398]
Surface modification	Fluoropolymer	A commercially polyurethane PICC catheter was modified by a three-step lamination process, with thin fluoropolymer layers to yield fluoropolymer–polyurethane–fluoropolymer composite structure before applying the liquid perfluorocarbon (LP)	*S. aureus* *S. epidermidis*	[337]
	*Hydrophobic polymer*			
Surface modification	Polytetrafluroethylene (PTFE)	SiO_2_ nanosphere was coated on PTFE catheter.	*E. coli* *S. aureus*	[399]

**Table 3:** The effectiveness of different antimicrobial agent coating and surface modifications approaches used in CA-UTIs prevention.

Strategy	Composite Used	Approach Used for Coating VC	Tested Microorganism	Reference
Release killing	*Metal*			
	Silver (Ag)	Silver–polytetrafluoroethylene (Ag-PTFE) nanocomposite coated UCs.	*E. coli* *P. mirabilis*	[409]
Release killing	copper ions (Cu)	Copper ions (Cu) and a polyphenol tannic acid (TA) were coated on urinary catheters (TA-Cu coated urinary catheters) using using one-step coordination method.	*E. coli* *P. mirabilis* *S. aureus*	[412]
Release killing	*Nanoparticles*			
	Silver (Ag-NPs)	Silver nanoparticles–polydopamine (AgNPs-PDA) coated catheters were designed.	*E. coli*	[413]
Release killing	Copper oxide (CuO-NPs)	Zn-doped CuO-NPs were coated on urinary catheters by sonochemical method.	*E. coli* *P. mirabilis* *S. aureus*	[414]
Release killing	Zinc oxide (ZnO NPs)	Zinc oxide nanoparticles (ZnO NPs) were decorated with amylase (biofilm matrix-degrading enzyme) by sonochemical method.	*E. coli* *S. aureus*	[415]
Release killing	*Antibiotics*			
	Chlorhexidine	Chlorhexidine-loaded poly(ε-caprolactone) nanospheres (CHX-NS) spray-adhered on urinary catheters.	*E. coli* *S. aureus*	[416]
Release killing	Chlorhexidine Triclosan	Chlorhexidine/Triclosan impregnated on silicone catheters.	*E. coli* *K. pneumoniae* *P. mirabilis* *E. feacalis*	[417]
Release killing	Sparfloxacin	Sparfloxacin-coated latex catheters using two immobilization methods.	*E. coli* *S. aureus*	[418]
	*Antimicrobial peptides*			
Contact killing	E6 (RRWRIVVIRVRRC)	A cysteine labeled peptide E6-coated polyurethane catheter was designed by covalent immobilization.	*P. aeruginosa* *S. aureus*	[419]
Contact killing	Chain201D (KWIVWRWRFKR) (from crowberry endophytes)	Chain201D coated on silicone surface model was designed by covalent immobilization.	*E. coli* *S. aureus*	[420]
Contact killing	Cys Lasio-III	Cys Lasio-III was immobilized on a commercial silicone catheter via a combination of AGE brush and PEG based chemical coupling.	*E. coli* *P. aeruginosa* *S. aureus* *En. faecalis*	[421]
	*Nitric oxide*			
Release killing	Nitrix oxide (NO)	NO-impregnated catheters were designed.	*E. coli*	[422]
	*Bacteriophages*			
Contact killing	The anti-*Pseudomonas* phage cocktail: ΦPaer4, ΦPaer14, M4, 109, ΦE2005-A, and ΦE2005-C The anti-*Proteus* phage cocktail: ΦPmir1, ΦPmir32, ΦPmir34, and ΦPmir37	Hydrogel-coated catheters were pretreated with phages.	*P. mirabilis* *P. aeruginosa*	[423]
Contact killing	The phage cocktail (podovirus vB_PmiP_5460 and myovirus vB_PmiM_5461)	Urinary catheters treated with phage cocktail were performed.	*P. mirabilis*	[33]
	*Hydrophilic polymer*			
Surface modification	Poly(N,N-dimethylacrylamide) (PDMAA)	The poly(N,N-dimethylacrylamide) (PDMAA) hydrogel coated on polyurethane ureteral stents.	*E. coli*	[424]
Surface modification	Poly(N,N-dimethylacrylamide) (PDMAA)	The hydrogel coating layer was formed using UV-crosslinking and swell-peeling methods.	*S. aureus*	[425]
Surface modification	Polydopamine/poly(N,N-dimethylacrylamide)	Polydopamine/poly(N,N-dimethylacrylamide)-coated silicone catheters (PDA/uhPDMA) using dip coating approach.	*P. aeruginosa*	[426]
Surface modification	Polyethylene glycol PEG	Silver-polyethylene glycol (mPEG-DOPA_3_) coated urinary catheters by cross-linking approach.	*E.coli*	[427]
Surface modification	Sulfobetaine methacrylate (SBMA)	Sulfobetaine methacrylate (SBMA) was grafted on silicone catheters using enzymatic approach.	*P. aeruginosa* *S. aureus*	[428]
Surface modification	Polytetrafluoroethylene PTFE	Silver- polytetrafluoroethylene (Ag-PTFE) nanocomposite grafted catheters were developed via a facile wet chemistry method.	*E. coli* *S. aureus*	[429]
	*Enzymes*			
Surface modification	Acylase	The immobilization of the enzyme on urinary catheters was done by layer-by-layer deposition technique.	*P. aeruginosa*	[430]
Surface modification	α-chymotrypsin (α-CT)	α-chymotrypsin (α-CT) covalently immobilized on low-density polyethylene surfaces (LDPE-α-CT).	*E. coli*	[431]
Surface modification	Glycoside hydrolases (Ghs)	PslGh modified surfaces using amine functionalization (APTMS) and glutaraldehyde (GDA)7 Linking.	*P. aeruginosa*	[432]
Surface modification	Cellobiose deshydrogenase (CDH)	CDH was covalently grafted onto plasma-activated urinary polydimethylsiloxane (PDMS) catheter surfaces.	*S. aureus*	[433]
